# Drusenoid Pigment Epithelial Detachment: Genetic and Clinical Characteristics

**DOI:** 10.3390/ijms22084074

**Published:** 2021-04-15

**Authors:** Taiyo Shijo, Yoichi Sakurada, Koji Tanaka, Akiko Miki, Seigo Yoneyama, Yumiko Machida, Aya Chubachi, Yu Wakatsuki, Atsushi Sugiyama, Hajime Onoe, Wataru Kikushima, Ryusaburo Mori, Kenji Kashiwagi

**Affiliations:** 1 Departments of Ophthalmology, Faculty of Medicine, University of Yamanashi, Kofu 400-8510, Japan; tshijoh@yamanashi.ac.jp (T.S.); syoneyama@yamanashi.ac.jp (S.Y.); asugiyama@yamanashi.ac.jp (A.S.); wkikushima@yamanashi.ac.jp (W.K.); kenjik@yamanashi.ac.jp (K.K.); 2 Department of Ophthalmology, Nihon University School of Medicine, Tokyo 173-8610, Japan; tanaka.koji@nihon-u.ac.jp (K.T.); machida.yumiko@nihon-u.ac.jp (Y.M.); wakatsuki.yu@nihon-u.ac.jp (Y.W.); onoe.hajime@nihon-u.ac.jp (H.O.); mori.ryusaburo@nihon-u.ac.jp (R.M.); 3 Department of Surgery, Division of Ophthalmology, Kobe University Graduate School of Medicine, Kobe 650-0017, Japan; acacyey@med.kobe-u.ac.jp (A.M.); achuba@med.kobe-u.ac.jp (A.C.)

**Keywords:** drusenoid pigment epithelial detachment, *ARMS2*, *CFH*, reticular pseudodrusen

## Abstract

Few studies report drusenoid pigment epithelial detachment (DPED) in Asians. In this multicenter study, we report the clinical and genetic characteristics of 76 patients with DPED, and, for comparison, 861 patients with exudative age-related macular degeneration (AMD) were included. On the initial presentation, the mean best-corrected visual acuity was 0.087 ± 0.17 (logMAR unit), and mean DPED height and width were 210 ± 132 and 1633 ± 1114 µm, respectively. Fifty-one (67%) patients showed macular neovascularization in the contralateral eye. The risk allele frequency of both *ARMS2* A69S and *CFH* I62V was significantly higher in DPED than in typical AMD and polypoidal choroidal vasculopathy (PCV) (*ARMS2* A69S risk allele frequency: DPED 77% vs. typical AMD 66% vs. PCV 57%, *CFH* I62V risk allele frequency: DPED 87% vs. typical AMD 73% vs. PCV 73%), although the risk allele frequency of both genes was similar between the DPED group and retinal angiomatous proliferation (RAP) group (*ARMS2* A69S: *p* = 0.32, *CFH* I62V, *p* = 0.11). The prevalence of reticular pseudodrusen (RPD) was highest in RAP (60%), followed by DPED (22%), typical AMD (20%), and PCV (2%). Although the prevalence of RPD differs between DPED and RAP, these entities share a similar genetic background in terms of *ARMS2* and *CFH* genes.

## 1. Introduction

Drusenoid pigment epithelial detachment (DPED) is characterized by a fairly well-circumscribed elevation of the retinal pigment epithelium and a confluence of drusen. The progressive growth and aggregation of large soft drusen is considered to be the origin of DPED, although its pathogenesis is not fully understood.

Casswell et al. described DPED as part of the clinical spectrum of non-exudative age-related macular degeneration (AMD) in 1985 [1]. A recent study using spectral-domain optical coherence tomography (SD-OCT) investigated the quantitative changes in PED and disruption patterns of retinal pigment epithelium (RPE) and Bruch’s membrane through the lifecycle of DPED and revealed that maximal volume, height, and diameters of DPED are correlated with visual prognosis [2]. 

Roquet et al. expanded on previous work and reported that progression to geographic atrophy (GA) or macular neovascularization (MNV) occurred within 2 years if the horizontal width of the DPED was greater than 2 disc diameters on initial presentation [3]. A report from the Age-related Eye Disease Study (AREDS) group found that the 5-year incidences of GA and MNV in the natural course of drusenoid PED were 19% and 23%, respectively [4].

To date, reports on DPED are almost exclusively seen in Caucasians, and there are few reports investigating the clinical and genetic characteristics of DPED in Asians. In this multicenter study, we genotyped *ARMS2* and *CFH* variants, two major variants associated with AMD, for patients with DPED and investigated the clinical characteristics of DPED at the initial presentation.

## 2. Results

A total of 937 patients, including 76 patients with DPED and 861 patients with exudative AMD, were enrolled in the present study. On the initial presentation, the mean best-corrected visual acuity was 0.087 ± 0.17 (logMAR unit), and mean DPED height and width were 210 ± 132 and 1633 ± 1114 µm, respectively. Of the 76 patients with DPED, genotyping was performed on 64. Table 1 shows the demographic and genetic characteristics of the participants at the initial presentation. The mean age of the DPED group was similar to that of the typical AMD group. Male distribution in the polypoidal choroidal vasculopathy (PCV) group was the highest among the four groups, followed by the rank order of the Drusenoid PED, typical AMD, and retinal angiomatous proliferation (RAP) groups. Prevalence of reticular pseudodrusen was highest in the RAP group among the four groups, followed by the rank order of the DPED, typical AMD, and PCV groups. The risk allele frequency of *ARMS2* A69S was significantly higher in the DPED group than in the typical AMD and PCV groups, although there was no significant difference in the risk allele frequency of *ARMS2* A69S between the DPED group and the RAP group. Similar to the risk allele frequency of ARMS2 A69S, the risk allele frequency of *CFH* I62V was significantly higher in the DPED group than in the typical AMD and PCV groups, although there was no significant difference in the risk allele frequency of *CFH* I62V between the DPED group and the RAP group.

Table 2 shows the demographic and genetic characteristics of DPED with or without reticular pseudodrusen at the initial presentation. Baseline DPED height was significantly higher in the RPD (−) group than in the RPD (+) group.

Table 3 shows the demographic and genetic characteristics of DPED between the MNV (+) and MNV (−) groups at the initial presentation. Baseline DPED height was significantly higher in the MNV (−) group than in the MNV (+) group.

Of the 51 patients with MNV in the contralateral eye, the prevalence of typical AMD, PCV, RAP, and scarring was 23 (45.1%), 9 (17.6%), 14 (27.5%), and 5 (9.8%), respectively.

## 3. Discussion

In this multicenter study, we reported the clinical and genetic characteristics of Japanese patients with DPED. To the best of our knowledge, this is the largest study investigating DPED in Asians.

Yu et al. recently reported that 42.5% of patients with AREDS2 were male and the mean age was 71.6 years [5]. In the present study, 55% were male and the mean age was 77.2 years. This might be due to racial differences, including retinal pigment epithelial pigmentation and integrity.

It has been reported that *ARMS2* and *CFH* are two major genes susceptible to age-related macular degeneration (AMD) through genome-wide association studies [6,7]. Several clinic-based studies have demonstrated that these genes are associated with exudative AMD, including typical AMD, polypoidal choroidal vasculopathy, and retinal angiomatous proliferation (RAP), although there have been no reports investigating the association between DPED and these genes [8,9]. In the present study, we compared the genetic characteristics of DPED with those of the three subtypes of exudative AMD and revealed that DPED and RAP share genetic characteristics in terms of *ARMS2* and *CFH.* Seddon et al. reported that the ARED severity scale increased by increasing the number of risk alleles in *ARMS2* and *CFH* [10]. DPED is a confluence of large soft drusen, and RAP exhibits large soft drusen in the macula [11]. Clinically, they share a fundus appearance. This might be one of the reasons why the risk allele frequencies of both genes were similar.

Reticular pseudodrusen (RPD), also known as subretinal drusenoid deposits, is a distinct entity from conventional drusen [12]. RPD has been reported to be associated with RAP and geographic atrophy. Alten et al. investigated 204 eyes with both PED and RPD and reported that the prevalence of DPED was 24% [13]. In the present study, RPD was observed in 17 (22%) of 76 patients with DPED. The prevalence was almost the same as that of typical AMD. To the best of our knowledge, this is the first report regarding the prevalence of RPD in eyes with DPED.

Of the 76 patients with DPED, 51 (67%) showed macular neovascularization (MNV) in the contralateral eye. However, there were no differences between the MNV (+) and MNV (−) groups, except for DPED height at the initial presentation. AREDS 2 did not report the prevalence of DPED depending on the absence or presence of MNV in the contralateral eye. Therefore, we could not compare the present results with those of other studies.

In the present study, we genotyped the ARMS2/CFH variants that were associated with inflammation of the RPE/choroid complex through complement pathway. However, the pathogenesis of AMD is various, such as angiogenesis, mitochondria, and ion-channel [14,15,16]. Further studies would be needed to clarify the different phenotypes among AMD patients.

The limitations of the study should be mentioned. The first limitation is that it was retrospective in nature; therefore, genotyping was not performed for all patients. The second limitation is that it was a cross-sectional analysis. In the current study, DPED height was lower in the RPD (+) group than in the RPD (−) group. A longitudinal study is necessary to confirm these results.

In summary, we described the clinical and genetic characteristics of DPED in Japanese patients. In particular, DPED and RAP share genetic backgrounds in terms of *ARMS2* and *CFH,* although the prevalence of RPD differs between the two entities.

## 4. Methods

### 4.1. Patients

The medical charts of consecutive patients with drusenoid pigment epithelial detachment at the University of Yamanashi Hospital, Nihon University Hospital, and Kobe University Hospital between January 2012 and December 2020 were used to compare the clinical and genetic characteristics between drusenoid PED and exudative AMD; patients with exudative AMD were enrolled at the University of Yamanashi between January 2012 and December 2020. The present study was approved by the institutional review board of each institute and was conducted in accordance with the tenets of the Declaration of Helsinki.

### 4.2. Diagnosis

At the initial presentation, all participants underwent a comprehensive ophthalmic examination, including measurement of best-corrected visual acuity (BCVA) using a Landolt chart, intraocular pressure (IOP), slit-lamp biomicroscopy with a +78-diopter (D) lens, color fundus photography, and spectral domain optical coherence tomography (SD-OCT) examination (Spectralis, Heidelberg Engineering, Dossenheim, Germany, HRA + OCT). If exudation was seen in the contralateral eye, fluorescein angiography (FA) and indocyanine green angiography (ICGA) were also performed.

**Figure 1:**
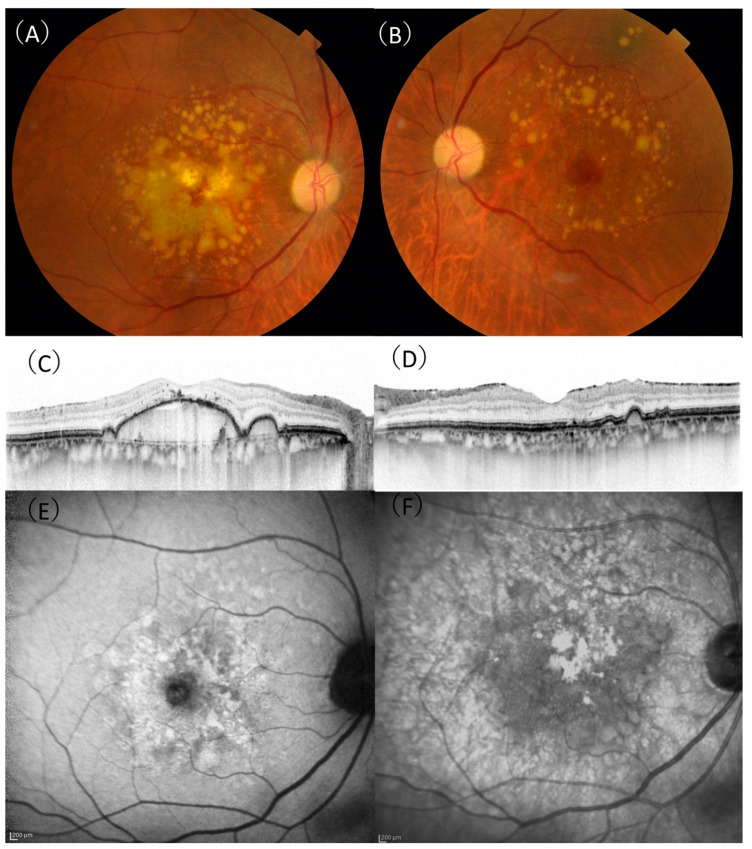
A 75-year-old male patient with drusenoid pigment epithelial detachment. (**A**) In the right eye, aggregation of confluent drusen was observed in the macula. (**B**) The aggregation of drusen in the left eye was spared from the central macula. (**C**) A horizontal optical coherence tomography (OCT) scan showing a large drusenoid pigment epithelial detachment with 307 µm height and 3943 µm width in the right eye. (**D**) A horizontal OCT scan showing retinal pigment elevation corresponding to a druse in the left eye. (**E**) There were no characteristic signs of reticular pseudodrusen on fundus autofluorescence. (**F**) There were no characteristic signs of reticular pseudodrusen on near-infrared reflectance.

**Figure 2:**
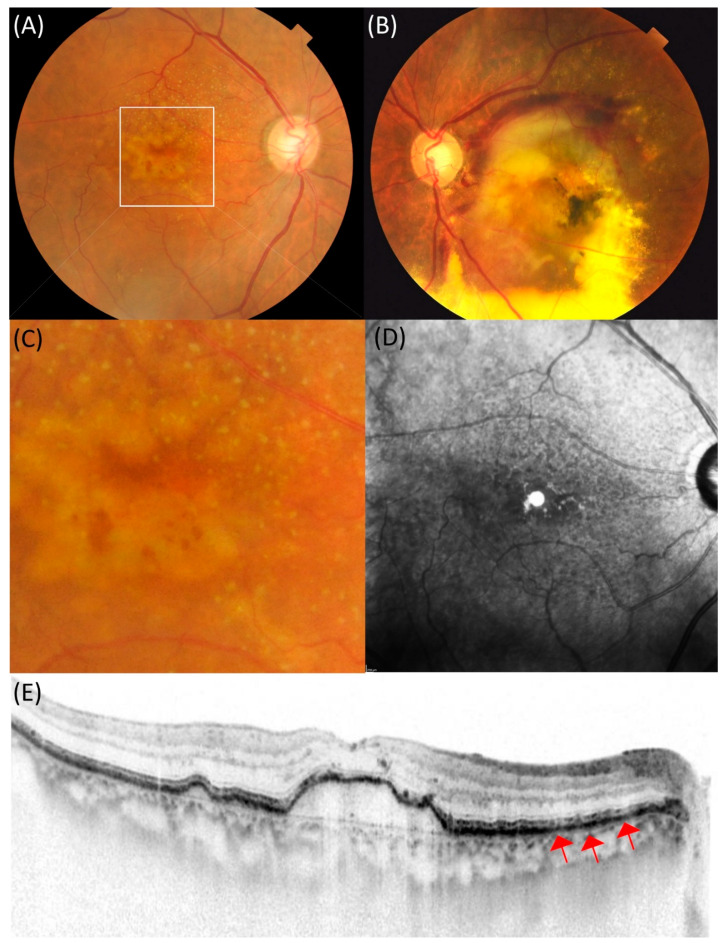
An 83-year-old female patient with drusenoid pigment epithelial detachment showing large subretinal hemorrhage in the contralateral eye. (**A**) In the right eye, drusen aggregation was observed in the macula. (**B**) In the left eye, the exudation, mainly subretinal hemorrhage, was seen in the macula. (**C**) Dot-type reticular pseudodrusen is seen in the magnified image of the macula in the right eye. (**D**) Hyporeflectance corresponding to the reticular pseudodrusen was mainly observed above the macula in the near-infrared reflectance. (**E**) Spectral-domain optical coherence tomography (SD-OCT) showed drusenoid pigment epithelial detachment with 113 µm height and width of 1872 µm in the macula. Red arrows indicate subretinal drusenoid deposits in the retinal pigment epithelium.

DPED was defined as a confluence of drusen of more than 500 µm in diameter and 100 µm in height on SD-OCT, as previously defined [17]. Depending on the presence or absence of MNV in the contralateral eye, patients with DPED were subdivided into the MNV (−) or MNV (+) groups. If there was no exudation in either eyes, these patients were included in the MNV (−) group. A representative case of the MNV (−) group is shown in Figure 1. If exudation occurred in the contralateral eye, these patients were included in the MNV (+) group. A representative case of the MNV (+) group is shown in Figure 2.

Exudative AMD was divided into three subtypes: typical neovascular AMD, PCV, and RAP, which were diagnosed based primarily on FA, ICGA, and SD-OCT findings. Eyes with typical neovascular AMD exhibited classic- or occult-type neovascularization in FA without polypoidal lesions on ICGA and SD-OCT findings of MNV either in the subretinal space or beneath the retinal pigment epithelium line. Eyes with PCV exhibited clusters of polypoidal dilation of the vessels with or without abnormal vascular networks in the superficial choroid in ICGA and irregularly elevated the RPE line on SD-OCT images. Eyes with RAP exhibited retinal–retinal or retinochoroidal anastomosis on FA or ICGA and retinal swelling with or without RPE detachment on SD-OCT, as previously reported [18].

The presence or absence of reticular pseudodrusen (RPD) was assessed using multimodal imaging, including color fundus photography, near-infrared reflectance, fundus autofluorescence, and SD-OCT. We defined the presence of RPD as confirmed by at least one imaging modality, as previously reported [19].

### 4.3. Genotyping

We genotyped two major variants, *ARMS2* A69S(rs10490924) and *CFH* I62V(rs800292), associated with AMD in the Japanese population. On initial presentation, peripheral blood was collected for genomic DNA analysis. The methods used to genotype variants of *ARMS2* A69S and *CFH* I62V have been previously described [20,21,22]. In detail, TaqMan genotyping assays contain sequence-specific primers to amplify the polymorphic sequence of the target genes and two minor groove binders to stabilize the samples. Purified wet genomic DNA was mixed with TaqMan genotyping assay and dispensed to a reaction plate, and the genotyping with real-time PCR system was performed. The allelic discrimination plot was collected and analyzed and recorded on an anonymous basis. Written informed consent was obtained from all patients.

### 4.4. Statistical Analysis

Statistical analyses were performed using SPSS (IBM, Tokyo, Japan). Best-corrected visual acuity measured on a decimal scale using a Landolt chart was converted into a logarithm of the minimal angle resolution (logMAR) for statistical analysis. The chi-square test was used to compare categorical variables between the two groups. The Mann–Whitney U test was used to compare continuous variables between the two groups. Statistical significance was set at *p* < 0.05.

## Figures and Tables

**Table 1:** Demographic and genetic characteristics of drusenoid pigment epithelial detachment and three subtypes of exudative age-related macular degeneration.

	DPED (*n* = 76)	tAMD (*n* = 328)	PCV (*n* = 476)	RAP (*n* = 57)
Mean age (years)	77.2 ± 7.0	76.6 ± 8.7	73.3 ± 8.2	82.5 ± 6.5
*p*-value (vs. DPED)		0.97	4.3 × 10^−4^	2.3 × 10^−5^
Gender: Male	42 (55.3%)	227 (69.2%)	359 (75.4%)	23 (40.4%)
*p*-value (vs. DPED)		0.02	0.0003	0.09
Presence of RPD	17 (22.4%)	65 (19.8%)	9 (1.9%)	34 (60.0%)
*p*-value (vs. DPED)		0.62	5.1 × 10^−15^	1.2 × 10^−5^
	**DPED (*n* = 64)**	**tAMD (*n* = 328)**	**PCV (*n* = 476)**	**RAP (*n* = 57)**
*ARMS2* A69S(c.205G > T) (rs10490924))				
TT	38 (59.4%)	152 (46.3%)	160 (33.6%)	39 (68.4%)
TG	23 (36.0%)	126 (38.4%)	223 (46.8%)	16 (28.1%)
GG	3 (4.6%)	50 (15.3%)	93 (19.6%)	2 (3.5%)
T-allele frequency	0.77	0.66	0.57	0.82
*p*-value (vs. DPED)		9.2 × 10^−3^	7.2 × 10^−5^	0.32
*CFH* I62V(c.184G > A) (rs800292)				
GG	48 (75.0%)	179 (54.6%)	259 (54.4%)	35 (61.4%)
GA	15 (23.4%)	122 (37.2%)	176 (37.0%)	20 (35.1%)
AA	1 (1.6%)	27 (8.2%)	41 (8.6%)	2 (3.5%)
G-allele frequency	0.87	0.73	0.73	0.79
*p*-value (vs. DPED)		0.0011	0.008	0.11

AMD: age-related macular degeneration; DPED: drusenoid pigment epithelial detachment; PCV: polypoidal choroidal vasculopathy; RAP: retinal angiomatous proliferation; RPD: reticular pseudodrusen.

**Table 2:** Comparison of demographic and genetic characteristics between drusenoid pigment epithelial detachment with or without reticular pseudodrusen.

	RPD(+) (*n* = 17)	RPD(−) (*n* = 59)	*p*-Value
Mean age (years)	77.65 ± 7.47	77.19 ± 6.96	0.78
Gender: Male	8 (47.1%)	34 (57.6%)	0.44
Baseline VA (LogMAR)	0.11 ± 0.22	0.082 ± 0.16	0.80
Presence of MNV in the contralateral eye	14 (82.4%)	37 (62.7%)	0.11
DPED height (µm)	151.12 ± 52.44	227.24 ± 142.49	0.028
DPED width (µm)	1339.77 ± 1182.09	1718.05 ± 1094.18	0.12
	**RPD(+) (*n* = 16)**	**RPD(−) (*n* = 48)**	***p*-Value**
*ARMS2* A69S(c.205G > T) (rs10490924)			
TT	12 (75.0%)	26 (54.2%)	
TG	4 (25.0%)	19 (39.6%)	
GG	0 (0.0%)	3 (6.2%)	
T-allele frequency	0.88	0.74	0.11
*CFH* I62V (c.184G > A) (rs800292)			
GG	12 (75.0%)	36 (75.0%)	
GA	3 (18.8%)	12 (25.0%)	
AA	1 (6.2%)	0 (0.0%)	
G-allele frequency	0.84	0.88	0.65

DPED: drusenoid pigment epithelial detachment; MNV: macular neovascularization; RPD: reticular pseudodrusen.

**Table 3:** Comparison of demographic and genetic characteristics between drusenoid pigment epithelial detachment with or without macular neovascularization in the contralateral eye.

	MNV(+) (*n* = 51)	MNV(−) (*n* = 25)	*p*-Value
Mean age (years)	77.43 ± 6.96	76.84 ± 7.30	0.74
Gender: Male	27 (53.0%)	15 (60.0%)	0.56
Baseline VA (LogMAR)	0.083 ± 0.18	0.095 ± 0.17	0.56
Presence of RPD	14 (27.5%)	3 (12%)	0.13
DPED height (µm)	183.50 ± 112.43	264.72 ± 152.12	0.0018
DPED width (µm)	1520.63 ± 1138.50	1863.56 ± 1058.77	0.086
	**MNV(+) (*n* = 48)**	**MNV(−) (*n* = 16)**	***p*-Value**
*ARMS2* A69S(c.205G > T) (rs10490924))			
TT	31 (64.6%)	7 (43.8%)	
TG	14 (29.2%)	9 (56.2%)	
GG	3 (6.2%)	0 (0.0%)	
T-allele frequency	0.79	0.72	0.39
*CFH* I62V(c.184G > A) (rs800292)			
GG	37 (77.1%)	11 (68.8%)	
GA	10 (20.8%)	5 (31.2%)	
AA	1 (2.1%)	0 (0.0%)	
G-allele frequency	0.88	0.84	0.65

DPED: drusenoid pigment epithelial detachment; MNV: macular neovascularization *n*.

## Data Availability

We will provide the data if necessary.
